# Robot-assisted gait training for balance and lower extremity function in patients with infratentorial stroke: a single-blinded randomized controlled trial

**DOI:** 10.1186/s12984-019-0553-5

**Published:** 2019-07-29

**Authors:** Ha Yeon Kim, Joon-Ho Shin, Sung Phil Yang, Min A. Shin, Stephanie Hyeyoung Lee

**Affiliations:** 1 Translational Research Center for Rehabilitation Robots, National Rehabilitation Center, Seoul, South Korea; 2 Department of Rehabilitation Medicine, National Rehabilitation Center, 58, Samgaksan-ro, Gangbuk-gu, Seoul, 01022 Republic of Korea

**Keywords:** Robot-assisted gait training, Stroke, Balance, Gait impairment

## Abstract

**Background:**

Balance impairments are common in patients with infratentorial stroke. Although robot-assisted gait training (RAGT) exerts positive effects on balance among patients with stroke, it remains unclear whether such training is superior to conventional physical therapy (CPT). Therefore, we aimed to investigate the effects of RAGT combined with CPT and compared them with the effects of CPT only on balance and lower extremity function among survivors of infratentorial stroke.

**Methods:**

This study was a single-blinded, randomized controlled trial with a crossover design conducted at a single rehabilitation hospital. Patients (*n* = 19; 16 men, three women; mean age: 47.4 ± 11.6 years) with infratentorial stroke were randomly allocated to either group A (4 weeks of RAGT+CPT, followed by 4 weeks of CPT+CPT) or group B (4 weeks of CPT+CPT followed by 4 weeks of RAGT+CPT). Changes in dynamic and static balance as indicated by Berg Balance Scale scores were regarded as the primary outcome measure. Outcome measures were evaluated for each participant at baseline and after each 4-week intervention period.

**Results:**

No significant differences in outcome-related variables were observed between group A and B at baseline. In addition, no significant time-by-group interactions were observed for any variables, indicating that intervention order had no effect on lower extremity function or balance. Significantly greater improvements in secondary functional outcomes such as lower extremity Fugl-Meyer assessment (FMA-LE) and scale for the assessment and rating of ataxia (SARA) were observed following the RAGT+CPT intervention than following the CPT+CPT intervention.

**Conclusion:**

RAGT produces clinically significant improvements in balance and lower extremity function in individuals with infratentorial stroke. Thus, RAGT may be useful for patients with balance impairments secondary to other pathologies.

**Trial registration:**

ClinicalTrials.gov Identifier NCT02680691. Registered 09 February 2016; retrospectively registered.

**Electronic supplementary material:**

The online version of this article (10.1186/s12984-019-0553-5) contains supplementary material, which is available to authorized users.

## Background

Balance impairments are common following stroke, often resulting in poor recovery of mobility and inability to perform activities of daily living unassisted. Such impairments represent a major risk factor for falls, which can lead to injury or death [1]. Although various interventions improve balance outcomes following stroke, no such intervention has been established as superior to others [2].

Balance impairments are a major concern in patients with infratentorial stroke, which accounts for 15–20% of all stroke cases [3, 4]. Infratentorial stroke is defined as stroke occurring below the tentorium cerebelli (including the cerebellum and brainstem), which is supplied by the vertebrobasilar artery. Common infratentorial posterior circulation symptoms include visual disturbance, vertigo, and ataxia [5]. The brainstem includes several cranial nuclei and relay pathways associated with eye movement, vestibular and somatosensory functions, and motor execution. The cerebellum plays a well-established role in modulating motor control, and brainstem dysfunction includes ataxia, dysdiadochokinesia, dysmetria, dysarthria, diplopia, and dysphagia [6, 7]. The cerebellum also contributes to the control of equilibrium and interlimb coordination during locomotion [8, 9]. A previous study suggests that the injury of infratentorial areas negatively affect balance functions [10]. Thus, balance impairments are common in patients with infratentorial stroke due to impaired integration of sensory information, postural control, and muscle strength.

Recently, the use of robot-assisted gait training (RAGT) for regaining and improving walking ability has increased among survivors of stroke [11]. During RAGT, the patient is placed in a supportive harness, and a robotic exoskeleton is attached to their lower extremities. The exoskeleton enables the application of guidance force provided by the robotic-orthosis during ambulation, thus allowing patients to engage in repeated practice of complex gait patterns at near-normal speed over a longer period. RAGT may enable practice enough to induce reorganization [12]. In conjunction with conventional physical therapy (CPT), it may result in significantly greater improvements in locomotor function than CPT alone [13].

In addition to providing both visual feedback and motor input for patients with stroke, RAGT is known to exert positive effects on balance [13–21]. However, it remains unclear whether RAGT results in greater improvements in balance than CPT [22]. While subsequent studies confirmed that RAGT results in favorable balance outcomes in patients with supratentorial stroke [23, 24], most studies have excluded patients with infratentorial stroke [16–21]. Because infratentorial stroke involves lesion in the cerebellum and the brainstem, it does not show the typical pattern of a supratentorial stroke. Indeed, to our knowledge, no studies have specifically investigated the effects of RAGT on balance among survivors of infratentorial stroke. Patients with infratentorial stroke often exhibit balance impairment because of eye movement impairment, vestibular functional deficits, vertigo, dizziness, and coordination deficits including ataxia, dysmetria, and dysdiadochokinesia other than motor weakness [25]. The infratentorial region including the cerebellum and brainstem is a pathway of the anterior corticospinal tract, tectospinal tract, vestibulospinal tract, and reticulospinal tract, which plays a role in balance; however, motor planning involving the supplementary motor area and premotor cortex is intact.

Therefore, this study aimed to compare the effects of RAGT combined with CPT and CPT only on balance function among survivors of infratentorial stroke. Given the lack of evidence regarding the use of RAGT in patients with infratentorial stroke, this study aimed to examine the effects of RAGT on balance function in this population. Our main hypothesis was that RAGT combined with CPT would produce clinically greater improvements in balance function than CPT only in individuals with infratentorial stroke.

## Methods

This single-blinded, randomized controlled trial with a crossover design was conducted at the National Rehabilitation Center in Korea. Participants were recruited from among patients who had been admitted to the stroke inpatient rehabilitation unit of the hospital from February 2015 to January 2017. Participants were still inpatients at the time of training. The inclusion criteria for this study were as follows: chief complaint of balance deficits rather than motor weakness following infratentorial stroke, no history of prior stroke, age > 19 years, and an absence of cognitive deficits that would interfere with the patient’s understanding and cooperation with instructions provided by the investigator (i.e., Mini-Mental State Examination score > 26). The exclusion criteria were as follows: contractures limiting range of motion in the lower extremities, lack of ambulation prior to stroke, severe cardiac disease, uncontrolled hypertension despite use of medication (average systolic blood pressure ≥ 140 mmHg or average diastolic blood pressure ≥ 90 mmHg measured over 7 days), presence of non-healing ulcers in the lower limbs, and osteoporosis. The experimental protocol was approved by the ethics committee of Korea National Rehabilitation Center (IRB no. NRC-2015-01-002).

Participants were randomly allocated to either group A or B using the NCSS-PASS program-generated randomization table, at an allocation ratio of 1:1. A principal investigator generated the random allocation sequence, a researcher enrolled participants, another researcher assigned participants to interventions, and a third-party blinded researcher assessed outcome measures. The randomization assignments were concealed in consecutively numbered, sealed opaque envelopes. The envelopes were opened sequentially after each patient provided written informed consent. Patients in group A underwent an intervention consisting of 4 weeks of RAGT combined with CPT, followed by 4 weeks of CPT. Patients in group B underwent the same interventions in reverse order (i.e., 4 weeks of CPT followed by 4 weeks of RAGT+CPT). The combined RAGT+CPT intervention consisted of 30 min of RAGT and 30 min of CPT (RAGT+CPT), while each intervention during the CPT only period consisted of 60 min of CPT (CPT+CPT). We referred to the former and latter as RAGT+CPT and CPT+CPT, respectively. Each intervention consisted of 20 sessions (five sessions each week). All participants were controlled for other gait-related treatments except RAGT and CPT provided in this study.

The Lokomat® robotic-orthosis (Hocoma AG, Zurich, Switzerland) system was used during RAGT. Participants were fitted with a harness so that a portion of their body weight could be supported when walking in the device. Typical initial body-weight support was provided at 70–80%. A minimum of 50% body-weight support was provided to allow participants to focus on the timing of their gait patterns. Typical initial walking speeds were approximately 1.0 km/h. Training difficulty was progressively increased by altering the walking speed and level of body-weight support. For each level of weight support, the speed of the robot-assisted gait was increased in increments of 0.2 km/h per session, up to 3.0 km/h. When the participant could ambulate at a certain level of body-weight support at the highest speed, the level of weight support was reduced by 5–10% per session to a lower limit of 50% (from 70 to 80%). The guidance force provided by the Lokomat was gradually reduced from 100 to 20%. The level of body-weight support and guidance force reduced simultaneously with patient compliance. The participants were requested to “walk with the robot.”

The primary goal of the CPT intervention was to facilitate improvements in static and dynamic balance. CPT consisted of balance-specific activities such as postural stability training, symmetric weight-bearing, general gait training, and trunk control. The structure of the intervention was customized to the functional capacity of each patient. All interventions were performed by skilled and experienced physical therapists. Two physical therapists kept training records that allowed comparison of similarity between therapists to minimize difference. After each intervention, therapists discussed and minimized the differences among the therapists on a daily basis. Each therapist worked with individuals in groups A and B.

Outcome measures were evaluated for each participant at baseline, after 20 intervention sessions (4 weeks), and after 20 sessions of the alternative intervention (8 weeks) by a blinded research therapist. Berg Balance Scale (BBS) scores, which are used to assess balance based on performance during 14 tasks representing common functional movements in daily life, were regarded as the primary outcome measure [26]. Each task is rated on a five-point scale (0–4), with the maximum score of 56 indicating that balance function is within the normal range. The BBS is a representative method for evaluating the balance ability of stroke patients [27]. The BBS measures both static and dynamic aspects of balance and has been demonstrated as good valid, good reliable, and fair to good responsiveness for use in patients with stroke [28–30].

Secondary outcome measures were as follows: (1) Static standing balance as measured using a force plate: Changes in center-of-pressure (COP) were measured to observe more detailed changes in static balance than those that could be captured using BBS scores. For tests of static standing balance, participants were instructed to stand on the force plate and focus on a centrally located spot in front of them, with arms hanging loosely by their sides. Each participant performed the following four tasks resembling those included in the Romberg test: standing with their feet positioned at shoulder-width under eyes-open (FSEO) or eyes-closed (FSEC) conditions, standing with their feet together with eyes-open (FTEO) or eyes-closed (FTEC). Each task was performed for 20 s and repeated three times. A 464 × 508-mm force plate (AMTI Force Platforms, Watertown, MA) was used to obtain a two-dimensional analysis of COP displacements along both the anteroposterior and mediolateral axes of the platform. The force plate was used to record the mean velocity of the COP displacement (mm/s) in the anteroposterior (COP Vel_AP_) and mediolateral (COP Vel_ML_) directions, as well as the area of the 90% confidence ellipse enclosing the COP (COP area in mm^2^). Data were processed and analyzed using Visual 3D™ software (C-Motion, Inc., Rockville, MD). (2) Trunk impairment scale (TIS) scores: The TIS was assessed because trunk control was related to standing balance [31]. The TIS was used to evaluate static sitting balance, dynamic sitting balance, trunk control, and coordination [32]. Scores range from 0 to 23 points, with higher scores indicating better sitting balance or trunk performance. Measures of trunk performance including the TIS have been significantly associated with measures of gait ability [33]. (3) Lower extremity Fugl-Meyer Assessment (FMA-LE) scores: The FMA-LE was assessed because improved balance may also contribute to lower extremity function. The 17-item FMA-LE was used to examine motor function and coordination of the affected lower extremity [34]. Total scores on the FMA-LE range from 0 to 34 points, with higher scores indicative of lower levels of impairment. Participants with quadriplegia were evaluated for the more affected side. (4) Functional Ambulation Category (FAC) scores: The FAC was used to assess gait ability, which was rated along six levels (scores ranging from 0 to 5) based on the amount of physical support required, regardless of whether an assistive device was used [35]. Balance function is a significant predictive factor for gait function [36]. A previous systematic review has indicated that balance and gait may share similar components that can be targeted using a single form of therapy [22]. Because gait is a comprehensive function that encompasses balance, RAGT should influence both gait and balance in this patient population. (5) Results of 10-m walk test (10MWT) at self-selected and fast walking speeds: The 10MWT was used to examine gait speed, and the participant was asked to walk on a 14-m walkway while wearing harness, under two conditions: at the fastest speed or at a self-selected, comfortable speed. Measurements were obtained for the 10-m region in the center of the walkway, while the 2-m acceleration and deceleration areas were excluded [37]. The 10MWT was performed three times, and the average value was used. The participants were using their own walking aid during 10MWT. (6) Falls Efficacy Scale (FES) scores: The FES requires participants to rank their confidence in their ability not to fall while performing various activities of daily living. The maximum score on the FES is 100 points [38]. (7) Scale for the Assessment and Rating of Ataxia (SARA) scores: The SARA was a clinical scale that is based on a quantitative assessment of cerebellar ataxia on an impairment level. Only three items related to balance were used: gait, stance, and sitting. A zero score means no ataxia, and a higher score means a more severe ataxia [39].

## Statistical analysis

The Kolmogorov-Smirnov test was used for normality test. The baseline homogeneity of groups A and B in BBS, static standing balance, TIS, FMA-LE, FAC, 10MWT, FES, and SARA was analyzed using the Mann–Whitney U-test. The potential effects of training order were examined by comparing results between groups A and B. Outcomes were thus compared using 2 (Group; group A, B) × 2 (Time; baseline, 8 weeks) repeated-measures analyses of variance (ANOVA).

Further comparisons were made for the effect of RAGT combined with CPT and CPT only on balance function among persons with infratentorial stroke. Differences between RAGT+CPT and CPT+CPT were compared using 2 (intervention: RAGT+CPT and CPT+CPT) × 2 (time: pre- and post-intervention) repeated-measures ANOVA. Post-hoc Wilcoxon signed-rank tests were used to compare pre- and post-intervention results between the RAGT+CPT and CPT+CPT interventions.

The likelihood ratio test was used to test the significance of the carry-over effect for treatment effect ($>{x}_{\mathrm{0.05,1}}^2=3.84$), using the following equation: *∆G*^2^ = (−2 *log L*_*Reduced*_) − (−2 *log L*_*Full*_) with *df*_*Full*_ *degrees of freedom*. Outcome variables displaying no carry-over effects were then compared using 2 (group: groups A, B) × 2 (time: baseline, 8 weeks) repeated-measures ANOVA.

All data were analyzed using SPSS version 20.0 for Windows (IBM SPSS Inc., Chicago, IL), and the level of significance was set at α = 0.05.

## Results

**Fig. 1:**
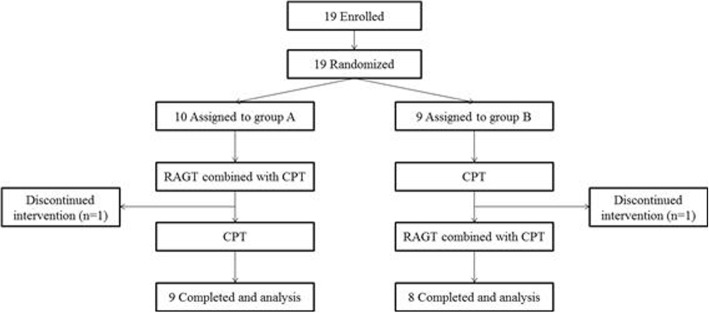
CONSORT flow diagram

Nineteen participants with infratentorial stroke (16 men, three women; mean ± standard deviation age: 47.4 ± 11.6 years) were included in this study (Tables 1 and 2). Participants were either in the subacute or chronic phases of post-stroke recovery, and the mean time after stroke was 15.3 ± 25.0 months. Of the 10 participants initially recruited in group A and the nine participants recruited in group B, two dropped out during the 4-week intervention, including one in group A (withdrew consent) and one in group B (withdrew consent). Ultimately, 17 participants (group A, *n* = 9; group B, *n* = 8) completed the study and were included in the final analysis (Fig. 1).

**Table 1:** Participant characteristics

No.	Sex	Age (year)	Height (cm)	Weight (kg)	Time since stroke (months)	FMA-LE score at baseline	SARA			Diagnosis
							Gait	Stance	Sitting	
Group A										
1	M	47	163	63	11	24	4	2	1	Quadriplegia d/t bilateral pontine infarction
2	F	39	165	70	111	27	4	3	0	Quadriplegia d/t bilateral cerebellar infarction
3	M	57	177	67	30	22	6	5	0	Quadriplegia d/t bilateral pontine and cerebellar infarction
4	M	56	169	75	30	28	2	2	0	Quadriplegia d/t bilateral midbrain and pontine hemorrhage
5	M	59	160	59	11	16	5	2	0	Quadriplegia d/t bilateral pontine infarction
6	M	44	170	71	3	31	2	2	0	Left hemiplegia d/t right medullary infarction
7	M	40	174	88	5	21	2	1	0	Right hemiplegia d/t left pontine hemorrhage
8	M	51	168	70	2	28	7	4	0	Right hemiplegia d/t right cerebellar hemorrhage
9	M	49	173	64	2	33	2	2	0	Right hemiplegia d/t right medullary infarction
10^a^	M	45	170	56	7	25	2	2	0	Right hemiplegia d/t Right cerebellar hemorrhage
Group B										
11	M	37	170	85	15	30	2	1	0	Quadriplegia d/t right pontine and cerebellar hemorrhage
12	M	67	171	77	3	21	5	1	0	Quadriplegia d/t left pontine and cerebellar infarction
13^a^	M	71	166	65	4	10	8	5	1	Quadriplegia d/t right hemiplegia d/t right cerebellar hemorrhage
14	F	33	165	62	2	28	5	3	0	Right hemiplegia d/t left medullary infarction
15	M	48	178	74	12	21	6	4	0	Right hemiplegia d/t left pontine hemorrhage
16	M	49	171	74	4	25	2	1	0	Quadriplegia d/t left pontine and bilateral cerebellar infarction
17	F	22	163	63	27	29	6	3	0	Quadriplegia d/t bilateral cerebellar hemorrhage
18	M	39	170	78	3	29	2	2	0	Quadriplegia d/t bilateral medullary infarction
19	M	48	167	63	8	29	5	4	0	Quadriplegia d/t bilateral cerebellar hemorrhage

^a^Two participants withdrew and were not included in the analyses. *FMA-LE* lower extremity Fugl-Meyer Assessment, *SARA* Scale for the Assessment and Rating of Ataxia

**Table 2:** Group demographics

		Group A	Group B	p
Sex	Male	9 (90.0%)	7 (77.8%)	0.466
	Female	1 (10.0%)	2 (22.2%)	
Age (year)		48.70 ± 7.01	46.00 ± 15.64	0.497
Height (cm)		168.90 ± 5.17	169.00 ± 4.42	0.968
Weight (kg)		68.30 ± 9.02	71.22 ± 8.24	0.497
Time since stroke (months)		21.20 ± 33.27	10.22 ± 8.54	0.842
FMA-LE score		25.50 ± 5.02	24.67 ± 6.50	0.905
SARA	Gait	3.60 ± 1.90	4.56 ± 2.13	0.356
	Stance	2.50 ± 1.20	2.67 ± 1.50	0.905
	Sitting	0.10 ± 0.32	0.11 ± 0.33	0.968

*FMA-LE* lower extremity Fugl-Meyer Assessment, *SARA* Scale for the Assessment and Rating of Ataxia

**Fig. 2:**
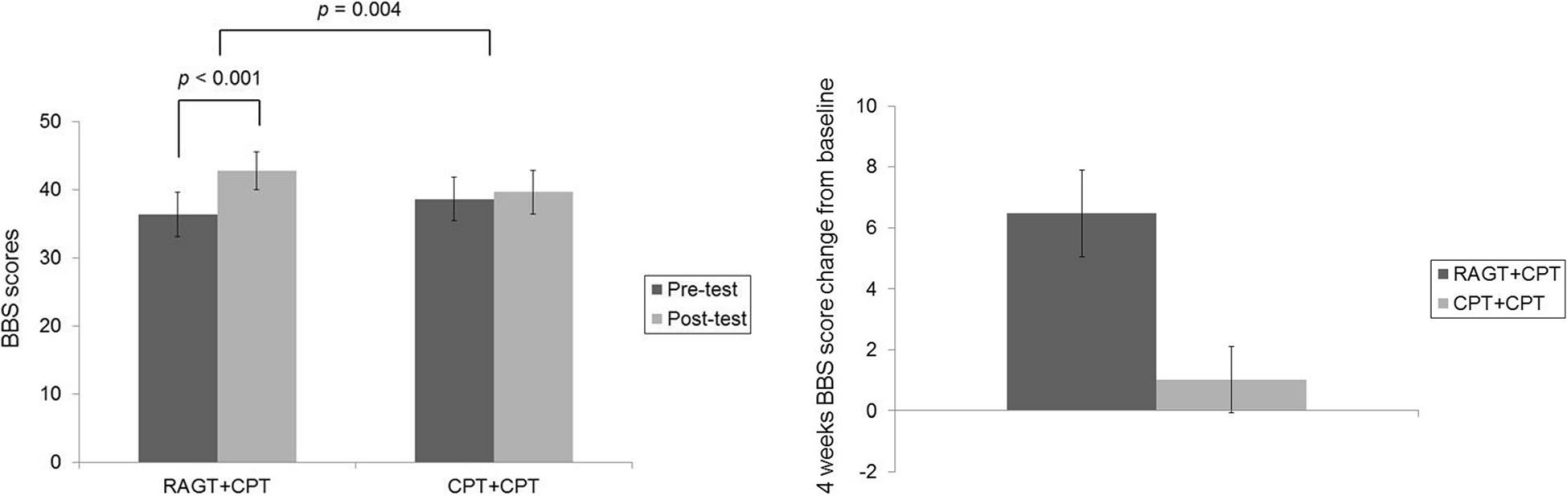
Left: BBS scores between RAGT+CPT and CPT+CPT groups before and after intervention. Right: Four weeks BBS score change from baseline. The error bars means standard errors. BBS: Berg Balance Scale; CPT: conventional physical therapy; RAGT: robot-assisted gait training

**Fig. 3:**
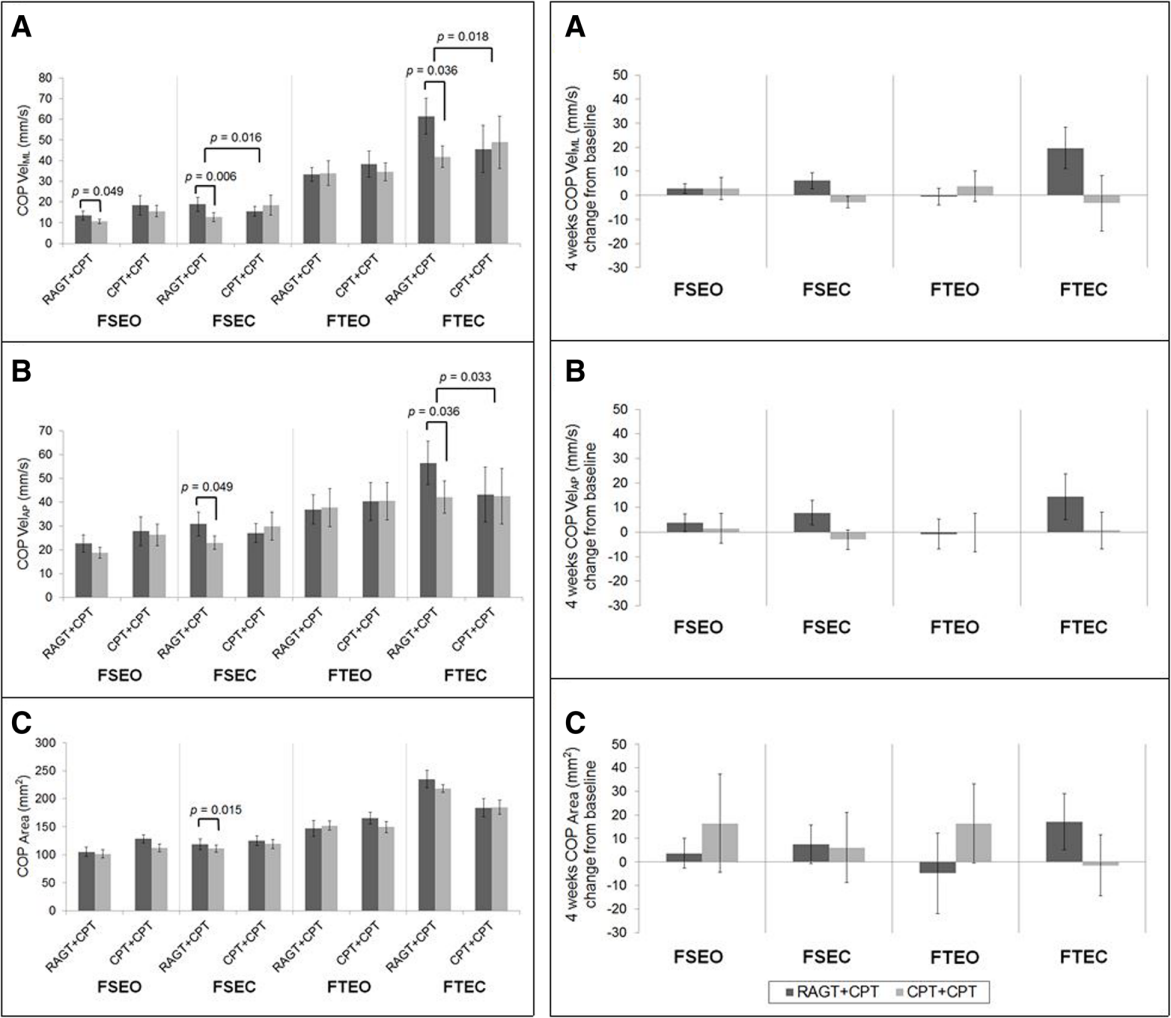
Left: COP-based variables during FSEO, FSEC, FTEO, and FTEC in the RAGT+CPT and CPT+CPT groups. Right: Four weeks COP-based variables change from baseline during FSEO, FSEC, FTEO, and FTEC. (A) COP Vel_ML_, (B) COP Vel_AP_, and (C) COP area. The error bars mean standard errors. COP: center of pressure; CPT: conventional physical therapy; FSEC: feet separated, eyes closed; FSEO: feet separated, eyes open; FTEC: feet together, eyes closed; FTEO: feet together, eyes open; RAGT: robot-assisted gait training; Vel_AP_: velocity in the anteroposterior direction; Vel_ML_: velocity in the mediolateral direction

Groups A and B exhibited no significant differences in outcome-related variables at baseline or time-by-group interactions (Table 3, Additional file 1: Figure S1 and Additional file 2: Figure S2). Therefore, findings were compared between the RAGT+CPT and CPT+CPT interventions, rather than between groups A and B. Significantly greater improvements in BBS scores were observed for RAGT+CPT than for CPT+CPT (F = 9.354, df = 1.000, *p =* 0.004) (Fig. 2). Figure 3 displays the changes in static standing balance throughout the intervention. Significantly greater improvements in COP Vel_ML_ during FSEC (*p* = 0.016) and during FTEC (*p* = 0.018) were observed for RAGT+CPT than for CPT+CPT. In addition, significant improvements were observed in COP Vel_ML_ during FSEO (*p* = 0.049), COP Vel_ML_ during FSEC (*p* = 0.006), COP Vel_ML_ during FTEC (*p* = 0.036)_,_ COP Vel_AP_ during FSEC *(p* = 0.049), COP Vel_AP_ during FTEC *(p* = 0.036), and COP area during FSEC *(p* = 0.015) for RAGT+CPT, but not for CPT+CPT. No significant differences or changes were observed in other variables associated with static standing balance.

**Table 3:** Outcome measures pre- and post-intervention in groups A and B

		Group A		Group B		F	P, time effect	P, group-by-time interaction	P, baseline comparisons
		Baseline	8 weeks	Baseline	8 weeks				
BBS		36.89 ± 14.74 40.00 (27.00–49.00)	43.11 ± 13.62 48.00 (35.00–53.00)	33.00 ± 11.99 34.00 (21.00–42.75)	41.88 ± 9.99 44.50 (31.50–50.75)	0.444	0.002	0.515	0.423
FSEO	COP Vel_ML_ (mm/s)	11.78 ± 7.78 10.47 (8.04–21.62)	14.07 ± 8.67 12.60 (7.10–27.70)	14.69 ± 5.38 14.11 (10.27–18.50)	9.96 ± 2.56 9.59 (7.74–11.83)	0.389	0.545	0.210	0.210
	COP Vel_AP_ (mm/s)	18.17 ± 11.90 16.27 (12.75–32.74)	21.17 ± 16.66 20.08 (11.17–53.22)	25.56 ± 13.29 20.31 (17.38–37.37)	19.35 ± 10.34 17.78 (10.60–24.02)	0.931	0.793	0.175	0.175
	COP Area (mm^2^)	99.13 ± 26.56102.95 (71.36–125.88)	105.65 ± 38.55103.11 (66.90–138.71)	146.28 ± 111.49108.45 (95.65–133.17)	89.05 ± 19.37 90.18 (69.79–107.19)	2.074	0.104	0.442	0.442
FSEC	COP Vel_ML_	12.64 ± 7.21 9.90 (7.00–19.66)	21.62 ± 30.27 8.28 (7.54–42.37)	15.09 ± 6.00 14.18 (9.43–21.23)	11.33 ± 3.37 10.64 (8.56–13.45)	2.537	0.527	0.657	0.657
	COP Vel_AP_	17.88 ± 5.20 18.30 (13.35–22.19)	31.29 ± 35.33 14.02 (12.65–58.57)	30.28 ± 15.61 27.93 (17.86–42.74)	26.12 ± 10.70 23.31 (16.84–34.60)	1.798	0.494	0.600	0.600
	COP Area	124.05 ± 49.10117.75 (92.09–155.72)	118.82 ± 59.80102.60 (82.21–150.58)	142.84 ± 78.93134.40 (71.68–218.84)	112.48 ± 55.39105.98 (81.52–115.11)	1.337	0.262	0.574	0.574
FTEO	COP Vel_ML_	39.85 ± 22.22 37.68 (17.42–60.22)	28.34 ± 13.00 24.82 (17.63–43.52)	46.24 ± 18.72 43.59 (29.42–64.39)	37.03 ± 18.07 41.21 (18.18–53.80)	0.020	0.239	0.573	0.573
	COP Vel_AP_	45.59 ± 27.43 46.26 (19.65–71.78)	37.46 ± 26.76 28.86 (16.61–63.59)	44.05 ± 21.02 41.73 (28.07–61.19)	40.22 ± 23.16 47.37 (16.40–60.47)	0.028	0.650	0.755	0.755
	COP Area	173.17 ± 71.37150.60 (114.56–246.49)	168.70 ± 73.69177.01 (89.24–234.05)	168.13 ± 77.28132.55 (104.89–249.15)	131.89 ± 39.47154.40 (89.65–162.88)	0.926	0.446	0.876	0.876
FTEC	COP Vel_ML_	46.05 ± 21.08 50.45 (23.12–56.45)	30.17 ± 22.01 19.84 (15.23–43.75)	69.20 ± 28.14 78.56 (39.36–89.67)	62.06 ± 22.64 62.54 (40.13–83.52)	0.176	0.320	0.286	0.286
	COP Vel_AP_	47.33 ± 29.39 41.97 (20.99–62.94)	23.18 ± 8.09 19.71 (17.40–19.71)	62.88 ± 25.69 68.35 (37.23–83.05)	50.27 ± 13.92 47.81 (38.45–64.55)	0.265	0.162	0.413	0.413
	COP Area	284.09 ± 217.10228.70 (112.50–363.87)	117.79 ± 19.68127.52 (103.87–121.03)	193.41 ± 67.05224.23 (116.49–246.71)	134.36 ± 15.12151.82 (125.63–164.56)	0.648	0.175	0.786	0.786
TIS		13.89 ± 4.37 14.00 (11.00–17.50)	15.44 ± 3.28 16.00 (13.00–18.00)	13.00 ± 3.16 13.50 (9.50–16.25)	17.62 ± 1.85 17.50 (16.25–18.75)	3.935	0.001	0.066	0.606
FMA-LE		25.56 ± 5.32 27.00 (21.50–29.50)	27.11 ± 3.92 28.00 (23.00–30.50)	26.50 ± 3.70 28.50 (22.00–29.00)	29.00 ± 3.07 29.00 (26.00–31.25)	0.422	0.014	0.526	0.673
FAC		3.00 ± 1.32 4.00 (1.50–4.00)	3.56 ± 1.33 4.00 (2.50–4.50)	2.50 ± 1.20 2.50 (1.25–3.75)	3.25 ± 0.71 3.00 (3.00–4.00)	0.205	0.008	0.657	0.423
10MWT (m/s)	Self-selected walking speed	0.49 ± 0.41 0.66 (0.07–0.88)	0.64 ± 0.42 0.76 (0.23–0.99)	0.25 ± 0.28 0.19 (0.02–0.49)	0.37 ± 0.32 0.26 (0.11–0.72)	0.033	0.067	0.858	0.333
	Fast walking speed	0.68 ± 0.59 0.87 (0.07–1.26)	0.97 ± 0.67 1.09 (0.26–1.48)	0.40 ± 0.40 0.22 (0.05–0.78)	0.51 ± 0.49 0.32 (0.11–1.09)	0.731	0.083	0.406	0.333
FES		59.44 ± 19.66 63.00 (46.50–75.00)	72.67 ± 25.52 79.00 (55.00–93.00)	45.88 ± 18.08 44.50 (32.50–59.00)	58.37 ± 13.36 57.50 (49.00–68.75)	0.006	0.012	0.937	0.139
SARA	Gait	3.78 ± 1.92 4.00 (2.00–5.50)	3.00 ± 1.58 2.00 (2.00–4.00)	4.13 ± 1.80 5.00 (2.00–5.75)	3.50 ± 1.78 3.00 (2.00–5.50)	0.050	0.057	0.825	0.650
	Stance	2.56 ± 1.24 2.00 (2.00–3.50)	1.89 ± 1.17 2.00 (1.00–3.00)	2.38 ± 1.30 2.50 (1.00–3.75)	2.00 ± 0.93 2.00 (1.00–3.00)	0.226	0.110	0.641	0.765
	Sitting	0.11 ± 0.33 0.00 (0.00–0.00)	0.00 ± 0.00 0.00 (0.00–0.00)	0.00 ± 0.00 0.00 (0.00–0.00)	0.00 ± 0.00 0.00 (0.00–0.00)	0.882	0.362	0.362	0.346

All data are presented as the mean ± standard deviation and median (interquartile range). *BBS* Berg Balance Scale, *COP* center of pressure, *FSEO* feet separated, eyes open, *FSEC* feet separated, eyes closed, *FTEO* feet together, eyes open, *FTEC* feet together, eyes closed, *TIS* Trunk Impairment Scale, *FMA-LE* lower extremity Fugl-Meyer Assessment, *FAC* Functional Ambulation Category, *10MWT* 10-m walking test, *FES* Falls Efficacy Scale, *SARA* Scale for the Assessment and Rating of Ataxia

Table 4 shows the results for the TIS, FMA-LE, FAC, 10MWT (self-selected walking speed and fast walking speed), FES, and SARA. Significant differences between the RAGT+CPT and CPT+CPT conditions were observed for FMA-LE (*p* = 0.001) scores and SARA gait (*p* = 0.033) and stance scores (*p* = 0.002), but not for TIS (*p* = 0.268), FAC (*p* = 0.140), FES (*p* = 0.062), or SARA sitting scores (*p* = 0.317). RAGT+CPT improved TIS, FMA-LE, FAC, FES, SARA gait, and SARA stance scores, while CPT+CPT improved TIS and SARA gait scores. Neither intervention significantly influenced 10MWT results for self-selected walking speed or fast walking speed.

**Table 4:** Secondary outcome measures pre- and post-intervention in the RAGT+CPT and CPT+CPT interventions

		RAGT+CPT			CPT+CPT			*P*, group-by-time interaction
		Pre-test	Post-test	*P, within*	Pre-test	Post-test	*P, within*	
TIS		14.35 ± 3.32 15.00 (12.00–16.50)	16.18 ± 3.36 17.00 (14.50–18.50)	0.003	14.00 ± 3.62 14.00 (10.50–17.00)	15.18 ± 2.58 15.00 (14.00–16.50)	0.040	0.268
FMA-LE		26.06 ± 4.41 28.00 (22.00–29.00)	28.41 ± 3.99 29.00 (25.00–32.00)	0.002	27.24 ± 4.24 28.00 (23.00–30.50)	26.88 ± 3.57 28.00 (23.00–29.50)	0.477	0.001
FAC		2.88 ± 1.17 3.00 (2.00–4.00)	3.41 ± 1.06 4.00 (3.00–4.00)	0.011	3.06 ± 1.35 3.00 (2.00–4.00)	3.18 ± 1.24 3.00 (2.00–4.00)	0.157	0.140
10MWT (m/s)	Self-selected speed	0.53 ± 0.31 0.62 (0.24–0.82)	0.61 ± 0.38 0.69 (0.18–0.86)	0.087	0.58 ± 0.39 0.64 (0.19–0.87)	0.59 ± 0.34 0.60 (0.31–0.80)	0.433	0.603
	Fastest speed	0.71 ± 0.47 0.87 (0.27–1.11)	0.96 ± 0.55 1.01 (0.45–1.26)	0.060	0.84 ± 0.57 0.81 (0.23–1.22)	0.85 ± 0.57 0.98 (0.36–1.23)	0.925	0.400
FES		56.88 ± 15.55 57.00 (49.50–65.00)	67.82 ± 21.36 71.00 (50.00–86.50)	0.018	61.94 ± 26.09 63.00 (39.50–86.50)	63.88 ± 21.41 62.00 (50.00–80.00)	0.629	0.150
SARA	Gait	3.76 ± 1.86 4.00 (2.00–5.50)	3.18 ± 1.63 2.00 (2.00–4.00)	0.020	3.94 ± 1.82 3.00 (2.00–5.00)	3.29 ± 1.72 2.00 (2.00–5.00)	0.039	0.033
	Stance	2.53 ± 1.18 2.00 (2.00–3.50)	1.76 ± 0.90 2.00 (1.00–2.50)	0.006	2.47 ± 1.23 2.00 (1.00–3.00)	2.00 ± 1.12 2.00 (1.00–3.00)	0.130	0.002
	Sitting	0.06 ± 0.24 0.00 (0.00–0.00)	0.06 ± 0.24 0.00 (0.00–0.00)	1.000	0.06 ± 0.24 0.00 (0.00–0.00)	0.06 ± 0.24 0.00 (0.00–0.00)	1.000	0.325

All data are presented as the mean ± standard deviation and median (interquartile range). *10MWT* 10-m walking test, *FAC* Functional Ambulation Category, *FES* Falls Efficacy Scale, *FMA-LE* lower extremity Fugl-Meyer Assessment, *CPT* conventional physical therapy, *RAGT* robot-assisted gait training, *SARA* Scale for the Assessment and Rating of Ataxia, *TIS* Trunk Impairment Scale

Significant carry-over effects were observed for all variables except FAC (G^2^ = 0.4) and SARA stance scores (G^2^ = 1.8). FAC (F = 12.775; *p* = 0.003), and SARA stance (F = 11.029; *p* = 0.005) scores exhibited significant group-by-time interactions, indicating that RAGT+CPT followed by CPT+CPT was superior with regard to independent walking and stance posture of ataxia.

## Discussion

This study is the first clinical trial to demonstrate the effect of RAGT on balance and lower extremity function among patients with infratentorial stroke. Our results indicated that RAGT+CPT resulted in significantly greater improvements in standing balance function than the same duration of CPT+CPT. In addition, improvements in balance confidence were observed following RAGT+CPT, but not following CPT+CPT.

Notably, BBS scores for the RAGT+CPT intervention increased by 6.5 points, exceeding the minimal detectable change of 6 points [40]. To our knowledge, only two studies have reported that RAGT is superior to CPT; however, they failed to achieve clinically meaningful changes in BBS scores [24, 40]. The subjects of Bang and Shin’s [24] study were patients who were already able to walk independently, so there might be a limit to the degree of improvement of balance ability. The treatment group of Yoshimoto et al. [41] underwent robot-assisted gait intervention once a week for 8 weeks (20 min/session), for a total of eight sessions, which may not be sufficient to achieve adequate improvement in balance. Conversely, in our study, RAGT facilitated clinically meaningful changes in balance function among individuals with infratentorial stroke. These results led to the improvement of SARA gait and stance scores in the RAGT+CPT group. This appears to be due to the SARA gait and stance evaluation reflects ataxia properties such as dysmetria and dysdiadochokinesia in individuals with infratentorial stroke.

When compared with CPT+CPT, the FMA-LE score significantly improved by only 2.35 points for RAGT+CPT. This improvement is below the most frequently used minimal clinically significant difference of 6 points recently suggested by Pandian et al. [42]. Similarly, the TIS score improved significantly but subclinically for both groups. Further studies involving larger numbers of patients are therefore required.

The observed effects of RAGT on balance can be explained by several possible mechanisms. First, RAGT may lead to somatosensory facilitation including proprioceptive systems, which should be emphasized among individuals with infratentorial stroke. Such enhancements would be marked in the eyes-closed condition, as patients with infratentorial stroke, who commonly exhibit vestibular or oculomotor dysfunction, may rely heavily on vision for maintaining balance. In our study, a significant difference between RAGT+CPT and CPT+CPT was observed for the eyes-closed condition only.

Second, RAGT enables loading and weight-shifting to the affected side, allowing for latero-lateral weight-shifting, resulting in symmetrical gait patterns [43, 44]. Because in quadriplegia weight is mainly supported by the less affected side, it is effective even if it is not hemiplegia. Similarly, RAGT+CPT exerted greater effects on COP Vel_ML_ than CPT+CPT, and the most prominent change due to RAGT+CPT was also observed for COP Vel_ML_. Thus, RAGT may allow for greater improvements in control over COP Vel_ML_, thereby decreasing the risk of falls [45].

Third, RAGT may have altered muscle activity in the lower extremities leading to improvements in functional performance. Partial weight-bearing gait training resulted in reduction in the mean burst amplitude of muscles for gastrocnemius and increase in mean burst amplitude of tibialis anterior [46]. These pattern changes resulted in better stability against common activation pattern in stroke patients, that is, higher activation of gastrocnemius and reduced activation of tibialis anterior. RAGT changed muscle coordination pattern with the controlling amount of weight support and stride frequency [47]. Moreover, the RAGT improved the motoneuronal firing rate by increasing motor unit firing without altering muscle force [48]. These muscle activity alterations without muscle force change may have led to an improvement in balance in patients with stroke who have difficulty in improving muscle strength.

In the present study, RAGT+CPT was more likely to result in better lower extremity function than CPT+CPT, as demonstrated by more significant improvement in FMA-LE scores. Within-group significant change was observed in FAC score of the RAGT+CPT group; however, no between-group difference was noted. Previous studies have reported that RAGT or treadmill-supported gait training resulted in greater improvements in lower extremity function compared with conventional gait training [22, 49, 50]. In addition, one systematic review reported that gait training may increase the risk of falls in older adults, while balance training may reduce this risk [51]. Indeed, RAGT may safely facilitate improvements in overall lower extremity function including balance.

However, no significant differences in sitting balance or trunk coordination as indicated by TIS scores were observed between RAGT+CPT and CPT+CPT. This finding suggests task-specific effects of RAGT on balance and biomechanically similar movements, in accordance with the findings of previous studies [52–54]. Our RAGT protocol may have been unable to recruit trunk stabilizers or improve trunk-related proprioception, as the trunk was equipped with a harness when the exoskeleton made large movements of the lower extremity.

On the contrary, most variables exhibited carry-over effects, except for FAC and SARA stance, for which we observed significant group-by-time interactions. Thus, RAGT+CPT followed by CPT+CPT was more effective with regard to independent walking and stance posture compared to intervention conducted in the reverse order. Previous researchers have proposed that proximal trunk control training prior to distal mobility training is essential for proper weight shifting and distal limb control [32]. Thus, prior RAGT may be optimal for improving independent gait and standing balance.

This study has some limitations. First, various evaluations related to balance, such as MiniBESTest or dynamic gait index, was not performed. In addition, we did not measure kinetic or electromyography data, which may be useful for determining the mechanisms underlying the effects of RAGT. This may result in insufficient understanding of the mechanism of RAGT. Second, in this study, the duration and intensity of RAGT could be too low to promote meaningful recovery, and the long-term effects of follow-up were not assessed. This did not confirm the underlying neuroplasticity based on impairment level. Third, the sample size was small, and this clinical trial was performed using a crossover design without a washout period. Moreover, the standard deviation of the static standing balance measure is large because of the gap between each subject’s balance ability, as this is a sensitive assessment. This might cause a type II statistical error. Therefore, there is a limit to the generalization of this result, and attention should be paid to statistical analysis.

## Conclusions

RAGT produced clinically significant improvements in static and dynamic balance and FMA-LE function in patients with infratentorial stroke. RAGT+CPT resulted in significantly greater improvements in standing balance and lower extremity motor function than the same duration of CPT. These findings indicate that RAGT may be useful for patients with balance impairments secondary to other pathologies, as infratentorial stroke shares many balance-related components.

## Additional files

Additional file 1: COP-based variables during FSEO, FSEC, FTEO, and FTEC from baseline to 8weeks in the groups A and B. (A) COP Vel_ML_, (B) COP Vel_AP_, and (C) COP area. The error bars means standard errors. COP: center of pressure; FSEC: feet separated, eyes closed; FSEO: feet separated, eyes open; FTEC: feet together, eyes closed; FTEO: feet together, eyes open; Vel_AP_: velocity in the anteroposterior direction; Vel_ML_: velocity in the mediolateral direction. (JPG 143 kb)

Additional file 2: Secondary outcome measures from baseline to 8weeks in the groups A and B. (A) TIS, (B) FMA-LE, (C) FAC, and (D) FES. The error bars means standard errors. FAC: Functional Ambulation Category; FES: Falls Efficacy Scale; FMA-LE: lower extremity Fugl-Meyer Assessment; TIS: Trunk Impairment Scale. (JPG 60 kb)

## Data Availability

Not Applicable.

## Abbreviations

10MWT: 10-m walk test
ANOVA: Analyses of variance
BBS: Berg Balance Scale
COP: Center of pressure
CPT: Conventional physical therapy
FAC: Functional Ambulation Category
FES: Falls Efficacy Scale
FMA-LE: Lower extremity Fugl-Meyer Assessment
FSEC: Feet separated, eyes closed
FSEO: Feet separated, eyes open
FTEC: Feet together, eyes closed
FTEO: Feet together, eyes open
RAGT: Robot-assisted gait training
SARA: Scale for the Assessment and Rating of Ataxia
TIS: Trunk Impairment Scale
VelAP: Velocity in the anteroposterior direction
VelML: Velocity in the mediolateral direction
