# Supplementary figures and images for: Robot-assisted gait training for balance and lower extremity function in patients with infratentorial stroke: a single-blinded randomized controlled trial
Source: J Neuroeng Rehabil. 2019 Jul 29;16:99. doi: 10.1186/s12984-019-0553-5 (PMC6664752; doi:10.1186/s12984-019-0553-5)

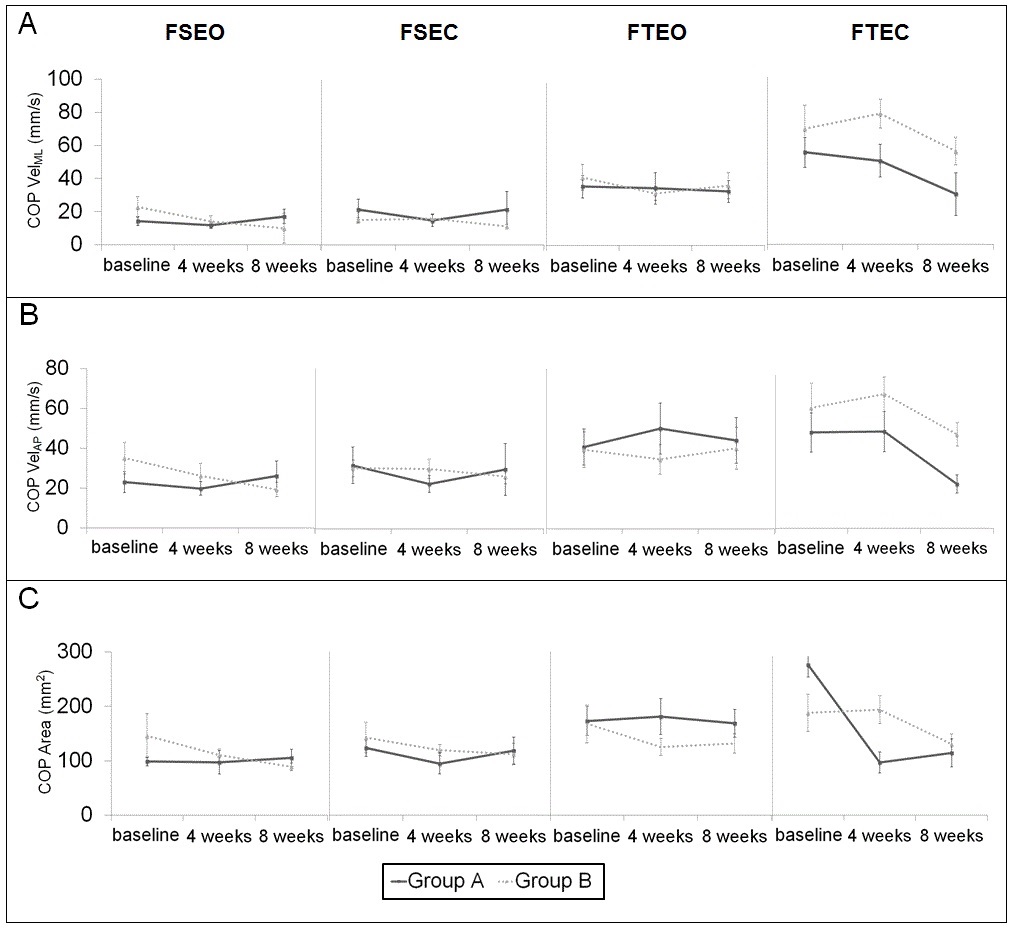

Supplement: Supplementary file 1 — COP-based variables during FSEO, FSEC, FTEO, and FTEC from baseline to 8weeks in the groups A and B. (A) COP VelML, (B) COP VelAP, and (C) COP area. The error bars means standard errors. COP: center of pressure; FSEC: feet separated, eyes closed; FSEO: feet separated, eyes open; FTEC: feet together, eyes closed; FTEO: feet together, eyes open; VelAP: velocity in the anteroposterior direction; VelML: velocity in the mediolateral direction. (JPG 143 kb) [file 12984_2019_553_MOESM1_ESM.jpg]

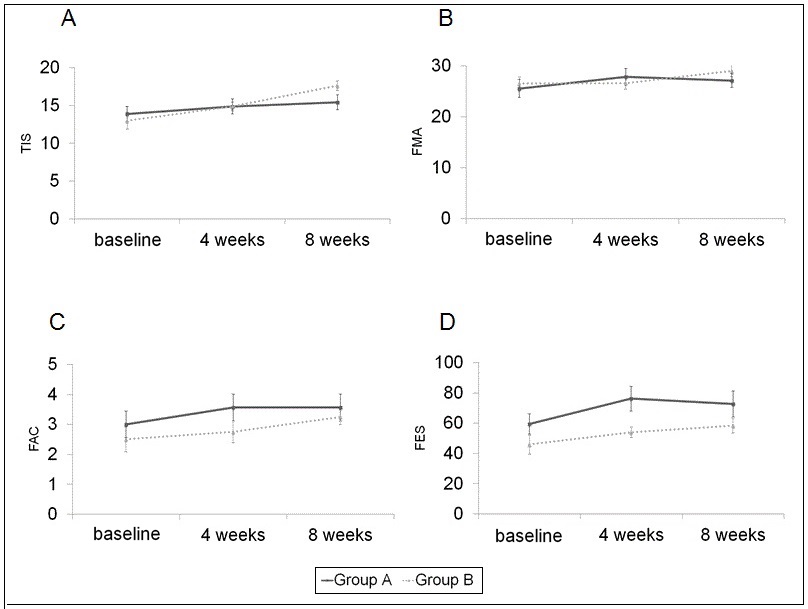

Supplement: Supplementary file 2 — Secondary outcome measures from baseline to 8weeks in the groups A and B. (A) TIS, (B) FMA-LE, (C) FAC, and (D) FES. The error bars means standard errors. FAC: Functional Ambulation Category; FES: Falls Efficacy Scale; FMA-LE: lower extremity Fugl-Meyer Assessment; TIS: Trunk Impairment Scale. (JPG 60 kb) [file 12984_2019_553_MOESM2_ESM.jpg]
